# Post‐Glacial Vegetation Trajectories on the Eastern Tibetan Plateau Reflect Millennial‐Scale Migration Lags in Complex Mountain Terrain Based on Sedimentary Ancient DNA and Dynamic Dispersal Modeling

**DOI:** 10.1002/ece3.70862

**Published:** 2025-01-22

**Authors:** Wei Shen, Stefan Kruse, Sisi Liu, Kathleen Stoof‐Leichsenring, Ingolf Kühn, Wenjia Li, Xianyong Cao, Zhi‐Rong Zhang, Chun‐Xia Zeng, Jun‐Bo Yang, De‐Zhu Li, Ulrike Herzschuh

**Affiliations:** ^1^ Alfred Wegener Institute, Helmholtz Centre for Polar and Marine Research, Polar Terrestrial Environmental Systems Potsdam Germany; ^2^ Institute of Environmental Science and Geography, University of Potsdam Potsdam Germany; ^3^ Department of Community Ecology Helmholtz Centre for Environmental Research – UFZ Halle Germany; ^4^ German Centre for Integrative Biodiversity Research (iDiv) Halle‐Jena‐Leipzig Leipzig Germany; ^5^ Geobotany and Botanical Garden Martin Luther University Halle‐Wittenberg Halle Germany; ^6^ Group of Alpine Paleoecology and Human Adaptation (ALPHA), state Key Laboratory of Tibetan Plateau Earth System, Environment and Resources (TPESER) Institute of Tibetan Plateau Research, Chinese Academy of Sciences Beijing China; ^7^ Germplasm Bank of Wild Species Kunming Institute of Botany, Chinese Academy of Sciences Kunming China; ^8^ Institute of Biochemistry and Biology, University of Potsdam Potsdam Germany

**Keywords:** ancient eDNA, connectivity, dynamic dispersal model, migration lag, refugia, trajectory analysis

## Abstract

Mountains with complex terrain and steep environmental gradients are biodiversity hotspots such as the eastern Tibetan Plateau (TP). However, it is generally assumed that mountain terrain plays a secondary role in plant species assembly on a millennial time‐scale compared to climate change. Here, we investigate plant richness and community changes during the last 18,000 years at two sites: Lake Naleng and Lake Ximen on the eastern TP with similar elevation and climatic conditions but contrasting terrain. We applied plant DNA metabarcoding to lake sediments leveraging a new regional reference database for taxa identification. Furthermore, we developed a simplified species dispersal model named SMARC. This was used to simulate species migration along river valleys in response to past climate change at the taxonomic resolution of the sedimentary ancient DNA (sedaDNA) approach. Statistical analyses, including ordination‐based ecological trajectory analysis, yielded a significant match between sedaDNA and simulated results at single taxon and community levels including certain site‐specific differences. Steep terrain downstream of Lake Naleng enhances connectivity to glacial lowland refugia during postglacial warming. In contrast, gentle terrain over long distances implies weak connectivity to the lowland and thus resulted in a strong migration lag at Lake Ximen. Likewise, terrain differences among our sites defined the different connectivity to alpine refugia during late‐Holocene cooling. Our consistent proxy‐ and model‐based results, for the first time, indicate that dispersal related migration lags in complex mountain terrain lead to uneven vegetation trajectories at sites with similar climatic conditions mainly because of differences in connectivity to refugia. Ultimately our results indicate that connectivity to refugia is a first‐order factor for species migration in addition to elevation‐related climatic conditions shaping the postglacial vegetation trajectory in mountainous terrain. This has hitherto largely been ignored when forecasting mountain vegetation responses to climate change and related risk assessment.

## Graphical abstract

This study investigates how mountain plant species on the eastern Tibetan Plateau responded to postglacial climate change, with a focus on the role of terrain‐driven connectivity to refugia. By combining sedaDNA metabarcoding and species dispersal modeling, we demonstrate that differences in connectivity to refugia led to distinct vegetation trajectories, providing critical insights into vegetation‐climate disequilibrium in complex mountain systems.

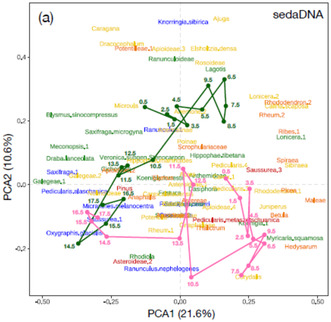

## 1 Introduction

Mountains with their complex terrain and steep environmental gradients, support diverse habitats and are widely recognized as biodiversity hotspots (Körner 2004). The Hengduan Mountains in the southeastern Tibetan Plateau (TP) are well known for their plant diversity (Sun et al. 2017; Liu et al. 2022). Due to their unique terrain, alpine regions in mountain systems are more sensitive to climate change compared to other terrestrial surfaces (Mountain Research Initiative EDW Working Group 2015). As global warming continues, rapid shifts are expected to result in upward migration of lowland species and regional loss of cold‐adapted alpine plants, posing a threat to mountain plant diversity (Thuiller et al. 2005). Therefore, understanding how species assembly along elevation gradients is crucial as it reveals the underlying mechanisms of biodiversity maintenance and provides effective conservation strategies for mountain ecosystems in response to climate change (Pavoine and Bonsall 2011).

Elevation, a surrogate for various environmental factors, is a critical variable to characterize the distribution of plant species in mountain regions (Oke and Thompson 2015). While it is generally assumed that species will shift upslope in response to climate warming, the migration process of plant taxa in mountainous terrain is far more complex and species‐specific. Empirical and experimental studies have revealed asynchronous rates and even directions of species range expansion and contraction (Alexander et al. 2018). For instance, it was proposed that “upslope movement” of an alpine meadow assemblage is faster than that of a high mountain forest assemblage (Breshears et al. 2008). In the Alps, nearly half of alpine species have failed to fill their potentially suitable ecological niches (Dullinger et al. 2012). The phenomenon is attributed to dispersal lag caused by complex mountainous terrain (Svenning and Sandel 2013). Multiple studies in Europe and Siberia have even revealed millennium‐scale vegetation‐climate disequilibrium (Herzschuh et al. 2016; Dallmeyer et al. 2022). However, the underlying processes, drivers, and temporalities that govern such compositional dynamics are still poorly understood, mainly because time‐series data for model validation are lacking.

The eastern TP is characterized by steep mountain valleys. They are assumed to serve as migration corridors (Rana et al. 2021) connecting lowland refugia with upland areas and thus helped shape species assembly during the Quaternary (Sun et al. 2017). Molecular phylogeographic studies of modern plant material have revealed three main patterns of upland‐lowland connectivity. First is the “contraction/recolonization” hypothesis (Meng et al. 2007) states that warm‐adapted species retreated to lowland refugia during glaciations and recolonized the Plateau during interglacial periods, resulting in expansion during warm period. Second is the “platform refugia/local expansion” hypothesis (Ma et al. 2014), suggests in situ survival of species on the upper TP during glaciations with restricted post‐glacial expansion (this is conceptually similar to the “microrefugia” hypothesis (Stewart et al. 2010), differing mainly in the scale of the refugia). In contrast to these postglacial expansions, the “Nunatak/sky island hypothesis” (Dahl 1987; He and Jiang 2014) describes a different pattern. It posits that cold‐adapted species expanded downward to lowland during glaciations, followed by a contraction to upland during interglacial periods. Unlike the first two hypotheses, which often exhibit clear phylogeographic structures reflecting post‐glacial expansion, species following the “Nunatak/sky island hypothesis” tend to have diffuse or undetectable phylogeographic patterns, likely due to their unique migration dynamics. Hence, the eastern TP is a promising region for studying long‐term vegetation migration, evaluating the impact of refugia connectivity on species assembly, and understanding the ecological trajectory under the influence of climatic changes.

Relying solely on modern genomic information or short‐term observations, however, is insufficient for accurately assessing long‐term migration processes. The mean temperature shift of about 4°C–7°C (Liu et al. 2014; Tierney et al. 2020) since the Last Glacial Maximum (LGM) marked a pivotal period for studying the response mechanisms of past vegetation and plant diversity to global warming. Numerous pollen‐based studies have utilized lake sediment archives to trace the post‐glacial vegetation history in the eastern TP (Kramer et al. 2010a, 2010b; Herzschuh et al. 2014) yielding a broadly similar pattern. The sparse alpine vegetation became alpine meadows at about 14 calibrated kilo‐annum before present (cal ka BP) followed by the encroachment of forests during the early Holocene, and ultimately forest retreat in the late Holocene. However, species assembly and how this is impacted by terrain has not yet been assessed. This is because of the limited taxonomic resolution of pollen records and the complexity of pollen source areas in mountain areas, characterized by strong uphill pollen transport (Herzschuh 2007). In contrast, several studies have shown that lake sedimentary ancient DNA (sedaDNA) predominantly reflects local or catchment‐scale vegetation signals (Parducci et al. 2017; Alsos et al. 2018; Jia et al. 2022). With advantages such as higher taxonomic resolution and the relative simplicity of its source, sedaDNA is increasingly recognized as the most suitable method for assessing vegetation biodiversity in terrestrial observatories (Liu et al. 2021; Jia et al. 2022).

Past, present, and future species distribution have been simulated using modern environmental factors and present‐day species occurrence data (Oke and Thompson 2015) to investigate the spatial patterns and dynamics of species on the TP (Rana et al. 2021; Yu et al. 2015). Most of these studies have utilized static niche‐based modeling methods (Guisan and Thuiller 2005), which are based on the assumption of equilibrium between species and climate (Alexander et al. 2018). Generally, only a few studies have incorporated migration into their modeling approaches (Dullinger et al. 2012; Meier et al. 2012; Alexander et al. 2018). River landscapes serve as natural corridors for vegetation migration, particularly in mountainous regions, where they create favorable conditions for the migration of lowland vegetation (Holeštová and Douda 2022). Consequently, migration along river valleys may have played a crucial role in the postglacial assembly of species along the steep mountain margins of the eastern TP (Schalow 1921). A comparative study between modeling and sedaDNA proxy data at the community level is still lacking.

This study aims to investigate how mountain plant species migrate in response to postglacial climate change and to what extent species migration is impacted by connectivity to upland and lowland refugia in mountain terrain. Here, we hypothesize that: (1) The impact on species migration increases with poorer connectivity to refugia, and (2) Steep terrain enhances connectivity to lowland refugia, whereas gentle terrain leads to longer distances to refugia, resulting in migration lag. To test these hypotheses, we selected two glacial lakes, Lake Naleng and Lake Ximen, with contrasting mountain terrain but generally similar climate conditions as research sites. Both alpine lakes have relatively small catchment areas, are situated near forest/meadow ecotones, and are highly sensitive to climate change, making them ideal for studying vegetation trajectories. We followed a sedaDNA plant metabarcoding approach along with a species dispersal modeling approach. This allows us to track the underlying drivers and processes of taxa richness and community compositional changes The quantitative understanding of mountain species assembly gained will be used to assess basic ecological principles including connectivity to refugia and any dispersal‐migrational lag at the community level. Our integrated data‐modeling approach evaluates the applicability of the “contraction/recolonization hypothesis”, “platform refugia/local expansion hypothesis” and “Nunatak/sky island hypothesis” in explaining eastern TP vegetation migration. These findings will provide a knowledge base for predicting taxa responses in the context of global change.

## 2 Material and Methods

### 2.1 Regional Setting

**FIGURE 1:**
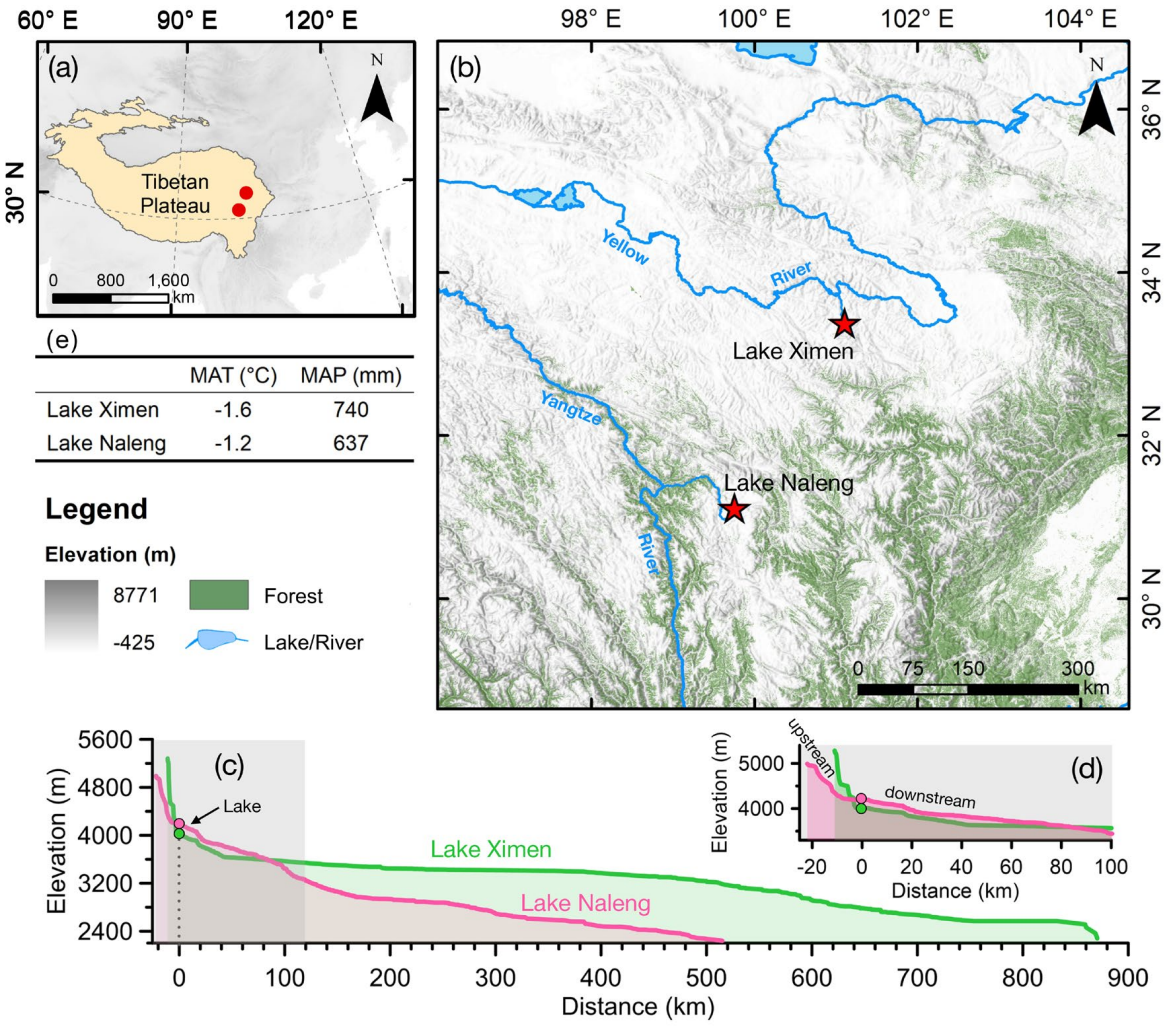
Study area in eastern Tibetan Plateau. (a) Locations of studied Lakes Ximen and Naleng. (b) Detailed map of the study area: Lacustrine core (red pentagram), river (blue), terrain elevation (grayscale), lowland forest (green fill). (c) Topographic profile of the catchments of Lakes Ximen and Naleng. The distance scales are relative to the lakes. Lake Naleng has a steeper downstream terrain (pink line) compared with Lake Ximen (green line). (d) Detailed view of the upper part of the topographic profile. The upland surrounding Lake Ximen (the upper part of which is covered by glacier/snow) is higher than that of Lake Naleng. (e) Estimates of present‐day mean annual temperature (MAT) and mean annual precipitation (MAP) for Lakes Ximen and Naleng.

The two study sites are located on the eastern TP, approximately 280 km apart (Figure 1). The Naleng Lake catchment in the Hengduan Mountains spans approximately 128 km^2^, with gently sloping terrain up to 4900 m above sea level (a.s.l.) but features extremely steep downstream topography. In contrast, the small catchment area of Lake Ximen, spanning approximately 53.3 km^2^, is dominated by the steep Nianbaoyeze Mountains, reaching elevations of up to 5369 m a.s.l. (with its upper part covered by glaciers and snow), while the lower terrain is relatively flat (Lehmkuhl, 1998). The summer, mainly controlled by the Indian monsoon and the East Asian monsoon, is warm and humid, and the winter, mainly controlled by the Westerlies, is cold and dry. Modern climate conditions are very similar at both sites (Figure 1e; Xu and Zhang 2017).

The vegetation at both sites shows generally similar elevation bands (Kramer et al. 2010a; Herzschuh et al. 2014). Shrubby vegetation composed of *Sibiraea*, *Spiraea*, *Lonicera*, *Salix*, *Rhododendron*, and *Potentilla* grows on shady slopes up to 4400 m a.s.l. in these areas. However, the catchment of Ximen lacks an arboreal layer, and the nearest relict stand of *Picea purpurea*, occurs 10 km away in the Lake Magen valley up to 4250 m, while Naleng has *Picea likiangensis* and *P. purpurea* distributed on north‐facing slopes in the catchment. High‐alpine meadows are widely distributed covering a wide elevation range from 3500 to 4500 m a.s.l., and are mainly composed of *Polygonum*, *Kobresia*, and Poaceae. In Ximen, sparse alpine vegetation characterized by cushion and rosette plants occurs on mountain slopes above 4800 m a.s.l., which is less well developed in the Naleng catchment.

### 2.2 Core and Subsampling

A sediment core of 12.81 m length was cored from the center of Lake Ximen (33.3792° N, 101.1035° E, 4000 m a.s.l.) in February 2004 (Zhang and Mischke 2009). The core was cut into 1 cm slices and transferred to the Alfred Wegener Institute for Polar and Marine Research (AWI) in Potsdam, Germany, where they were stored in the cool room at 4°C. The inner part of 135 core sediment samples were subsampled at a 150‐year interval, based on the published age‐depth model (Herzschuh et al. 2014). Prior to subsampling, the work surface and tools were cleaned with DNA ExitusPlus (VWR, Germany) and distilled water, then irradiated with UV light to decontaminate the surfaces in the working hood. Clean subsampling equipment was used to remove a thin layer of the sediment surface by using disposable sterile scalpels. For further processing, only the inner part of the sediments was used. The subsampling environment was under strict hygienic rules to prevent contamination from modern DNA. In total, 135 core sediment samples were collected into 8 mL sterile tubes (Sarstedt) and stored at −20°C until further use.

### 2.3 DNA Extraction and Amplification

DNA extraction and pre‐PCR steps were performed in the paleogenetic laboratories at AWI Potsdam, Germany, using strict ancient DNA precautions and protocols (Epp, Zimmermann, and Stoof‐Leichsenring 2019). DNA was extracted with the PowerMaxSoil DNA Isolation kit (Mo Bio Laboratories Inc., USA) using approximately 1.0 g of sediment. Each of the 15 extraction batches taken contained 9 samples and an extraction blank control. After extraction, DNA concentration was measured with a Qubit 2.0 Fluorometer (Invitrogen, USA) and samples were diluted to 3 ng/μL. For amplification, we used the plant universal primers g and h targeting the P6 loop of the chloroplast *trn*L (UAA) intron, which is the most commonly used DNA marker for vascular plants (Taberlet et al. 2007). Polymerase chain reaction (PCR) was performed on three replicates although only two were used in this study to facilitate comparison to the data from Lake Naleng which only had two PCR replicates. Each PCR reaction contained 3 μL sedaDNA template, 0.4 μM, 1× Platinum Taq DNA Polymerase High Fidelity PCR buffer (Invitrogen, USA), 1 U Platinum Taq High Fidelity DNA Polymerase (Invitrogen, USA), 0.25 mM dNTPS, 0.8 mg Bovine Serum Albumin, 2 mM MgSO_4_ (Invitrogen, USA) and Primers (forward: 5′ NNN(8 bp tag)GGGCAATCCTGAGCCAA 3′, reverse: 5′ NNN(8 bp tag)CCATTGAGTCTCTGCACCTATC 3′). Each PCR batch includes nine DNA extractions, one extraction control and No Template Control (NTC). If the negative control fails, the PCR is repeated until a negative control is achieved. PCRs were performed in a PCR thermocycler separated from the ancient DNA facilities in a post‐PCR area. The PCR protocol involves an initial denaturation step at 94°C for 5 min, followed by 40 cycles consisting of denaturation at 94°C for 30 s, annealing at 50°C for 30 s, extension at 68°C for 30 s, and a final extension step at 72°C for 10 min. Further details about the steps of DNA extraction, PCR set‐up, amplification, PCR product purification, and pooling are presented in Liu et al. (2021). Next‐generation sequencing was conducted on an Illumina NextSeq 500 sequencing platform (2 × 150 bp, NextSeq Mid kit) at Genesupport Fasteris SA, Switzerland. Our project was sequenced together with three other sequencing projects (using different index primers). The sequencing of our project resulted in 8.2 Gb with 27,307,497 generated clusters with a quality of 85.19% ≥ Q30. The data for Lake Naleng came from Liu et al. (2021) and included 71 samples that were treated in the same manner.

### 2.4 Reference Database

Taxonomic classification was facilitated by a customized East Tibetan reference database containing 309 unique amplicon sequence variants (ASVs) derived from 911 specimens representing 890 species (Supporting Information). The database includes species naturally distributed in eastern Tibet at elevations of at least 3000 m a.s.l. To create the reference database, the P6 loop of the *trn*L (UAA) intron was extracted from chloroplast whole genomes assembled through low‐coverage genome skimming conducted at the Kunming Institute of Botany, Chinese Academy of Sciences. Subsequently, we constructed the custom reference database using ecoPCR in OBITools package (Boyer et al. 2016) that involves an in *silico* amplification with the plant universal primers g and h targeting the P6 loop of the chloroplast *trnL* (UAA) intron (Taberlet et al. 2007). Finally, the database was formatted for use with OBITools using the *build_ref_db* function. For simplicity, ASVs are hereafter referred to as taxa.

### 2.5 Sequence Analysis

Quality filtering, demultiplexing, and taxonomic classification were performed with the OBITools package (Boyer et al. 2016) using the steps: Illuminapairedend (aligning read pairs) ngsfilter (assigning sequence reads to samples), obiuniq (dereplicate sequences), obiclean (exclude potential PCR or sequencing errors), and ecotag (taxonomic classification against the Kunming dataset). Only those matches that showed a 100% identity to the reference database (assignment to at least family level), were retained and only when they were present in at least two PCR replicates or samples. The extraction blanks and PCR no template controls (NTC) were mostly without any contamination (0.1% of read counts from total read counts). Only a few Saliceae reads (0.05% of total Saliceae counts in all samples and blanks) and Nolinoideae (3 blank outliers) were identified.

### 2.6 Modeling Approach

We aim to explore the impact of mountainous terrain on post‐glacial vegetation trajectories and test the hypothesis about the impact of dispersal lags on the colonization of plants in a lake catchment and hence the observable biodiversity. To achieve this, we set up a simplified species migration along river corridors (SMARC) model (see a detailed overview following the ODD protocol, Text S2 of Appendix S1) (Grimm et al. 2006, 2010). SMARC considers the observed elevation distribution range of a taxon under contemporary climate. Using elevation as the predictor for potentially suitable conditions in the past, the occupation of this environmental space is realized with a certain probability and can additionally be constrained by dispersal limitation, which may lead to a migration lag (Svenning, Normand, and Skov 2008).

The simulation approach was set up for species groups (for simplification called taxa) reflecting those with a similar amplicon sequence variant to the sedaDNA approach. We simulated the elevation range distributions of plant taxa for the time period since 18 cal ka BP. In the “Elevation‐only” mode, there are no dispersal limitation. In the second “Terrain” mode, dispersal limitation along river corridors is considered, with seed dispersal limitation set at 100 m/year (Svenning and Sandel 2013). For simplification, complex diffusion patterns and differences among species are ignored.

For ease of comparison, we consider only those taxa shared by the two lakes Ximen and Naleng for modeling. We collected modern elevation distribution data of species potentially occurring in the lakes region to provide necessary input data for each taxon on the level of proxy data. Elevation distribution data for plant species was collected from the database of Flora of China (www.iplant.cn, accessed March 8, 2022). For each of the 126 taxon sequences, which correspond to one or more species, we calculated mean elevation values based on their known distribution ranges.

Terrain profiles along the river were extracted from the 90‐m resolution SRTM digital elevation model (Jarvis et al. 2008). For climate forcing, we matched the reconstructed (based on δ^18^O) high‐resolution and variable temperature record from Dongge Cave to fit with the coarsely reconstructed temperature anomaly curve for the Northern Hemisphere (30°–90° N) since the last deglaciation (Dykoski et al. 2005; Shakun et al. 2012). To allow the taxa to reach equilibrium with the climate data at the beginning of the series, the temperature anomaly for 21.5 cal ka BP was applied to all years back to 50 cal ka BP. Before running the model, the climate and terrain data were linearly interpolated to make the data regular with a specified time step of 25 years and a distance step of 250 m along rivers.

The SMARC simulations have the following sequence at each time step: (1) Calculate the potential ecological niche distribution of the taxa at that time by applying the temperature lapse rate of 0.55°C/100 m (Li et al. 2013) to the temperature anomaly. (2) Simulate taxa colonization and retreat along the terrain. When a taxon is not yet present it can establish in the terrain with a probability of 80% and colonizes the lowest potential position with a 1% chance. Without seed dispersal limitation in the “Elevation‐only” mode, the potential cells can be colonized with a chance of 80%. Alternatively, in the “Terrain” mode, a dispersal limitation can be set to 100 m/year (Svenning and Sandel 2013, Snell and Cowling 2015, Tiebel et al. 2020), ignoring complex diffusion patterns and differences among species, for simplification. When a taxon has colonized an area, the probability of it reaching novel positions depends on its median distributional breadth (calculated by the absolute range divided by the median of observations). The colonization probability is moderated by assuming that dispersal ability increases with the number of currently colonized positions (qualitatively for > 1 and ≤ 10 positions, probability is doubled and for > 10 positions, it becomes three times more likely). If it advances, it reaches at least one step in elevation toward the maximum distance. The same procedure applies for retreating to lower elevations. Extinction for environmental conditions outside the species' niche is harsh at both edges. (3) Compute the presence and absence of taxa in the catchment of the lake. If the upper limit of the simulated distribution range of a considered taxon is above the lake elevation, we use 1 to represent presence in the catchment and 0 if it is absent.

We repeated the simulations 30 times for each lake. We restricted model evaluation to those 74 taxa with an abundance > 0.03% in the sedaDNA proxy because rare taxa or taxa with low biomass do not yield a reliable signal in the proxy record. The similarity and correlations are calculated using the simple matching coefficient and corresponding permutation test. We conducted a sensitivity analysis to assess the robustness of the findings to various modeling parameters. We find that simulation results are relatively robust to parameter changes for mid‐to‐lowland taxa, which exist in a relatively wide range of niches, and will not easily go extinct in the regional environment.

For a more detailed description of the protocol, sensitivity analyses, and workflow of the SMARC model, see the Text S2, S3, S4 of Appendix S1.

### 2.7 Statistical Analyses

All statistical analyses were performed in R 4.1.0 (R Core Team 2021). To allow a comparison of Ximen sedaDNA records with Naleng, and to obtain normalized count data, we rarefied the data based on the minimum number of counts (6652) 100 times. Both rarefied datasets were averaged in one‐thousand‐year increments to achieve the same temporal resolution. Before analyzing the vegetation trajectory of changes, a Box‐Cox‐chord transformation with exponent 0 was applied to the community compositional data to reduce the skewness of many zeros and obtain a double‐zero asymmetrical matrix (Legendre and Borcard 2018). Species were grouped according to their mean maximum elevation value, and changes in richness for the different subgroups were estimated to show trends in the taxa within different biotopes. Principal component analysis (PCA) was used to plot community trajectory changes, using the ‘rda’ function of the “vegan” package (Oksanen et al. 2022).

All statistical analyses were performed in R 4.1.0 (R Core Team 2021). To allow a comparison of Ximen sedaDNA records with Naleng, and to obtain normalized count data, we rarefied the data based on the minimum number of counts (6652) 100 times (https://github.com/StefanKruse/R_Rarefaction). The mean values of the 100 resampled data were used for further analysis. To facilitate a visual comparison of how taxa change through time in different records, the ‘strat.plot’ function from the “rioja” package (Juggins 2020) was used to produce barplots and the stratigraphic diagram for all shared taxa. A constrained hierarchical cluster analysis was performed with the ‘dist’ and ‘chclust’ functions by sample depth (Grimm 1987). Both rarefied datasets were averaged in one‐thousand‐year increments to achieve the same temporal resolution. Before analyzing the vegetation trajectory of changes, a Box‐Cox‐chord transformation with exponent 0 was applied to the community compositional data to reduce the skewness of many zeros and obtain a double‐zero asymmetrical matrix (Legendre and Borcard 2018). Species were grouped according to their mean maximum elevation value, and changes in richness for the different subgroups were estimated to show trends in the taxa within different biotopes. Principal component analysis (PCA) was used to plot community trajectory changes, using the ‘rda’ function of the “vegan” package (Oksanen et al. 2022). The Pearson correlations of PCA1 scores and trajectory segments between simulation and proxy were computed using the ‘cor.test’ function in the “stats” package (www.r‐project.org). The similarity and correlations of taxa between the sedaDNA and SMARC model are calculated using the simple matching coefficient (SMC) and corresponding permutation test.

## 3 Results

### 3.1 SedaDNA Plant Metabarcoding From Lake Ximen (New) Compared to Lake Naleng

After bioinformatic filtering of the raw sequencing data, 13,648,770 and 5,405,425 sequence counts were obtained from the 135 (270 PCR replicates) and 71 (138 PCR replicates) sediment samples from Lakes Ximen and Naleng, respectively. Aligning the sequences with the East Tibetan reference database resulted in 192 terrestrial Spermatophyta amplicon sequence variants (for simplification here called taxa) belonging to 46 families with a 100% best‐match identity (Appendix S2). Among them, 30 taxa were identified at the family and tribe level, 113 taxa at the genus level, and 49 taxa at the species level.

**FIGURE 2:**
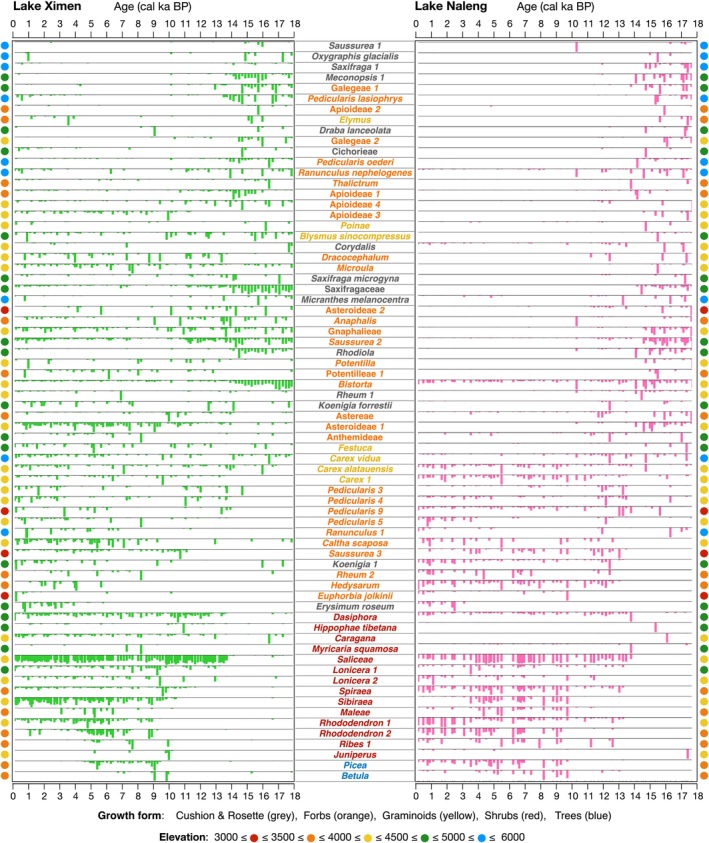
Stratigraphic plots showing the relative abundance (%) of terrestrial plant sedaDNA for shared taxa from Lakes Ximen and Naleng. Only shared taxa with 100% match to a customized reference database, frequency > 3 (present in at least 3 samples) and abundance > 0.02% in each lake are shown. The taxa are shown on sequence level. Taxa names are colored as follows: Cushion and rosette taxa (gray), forbs (orange), graminoids (yellow), shrubs (red), and trees (blue). The dots between the stratigraphic plots represent the mean value of the species elevation maximum in the species list corresponding to sequences.

**FIGURE 3:**
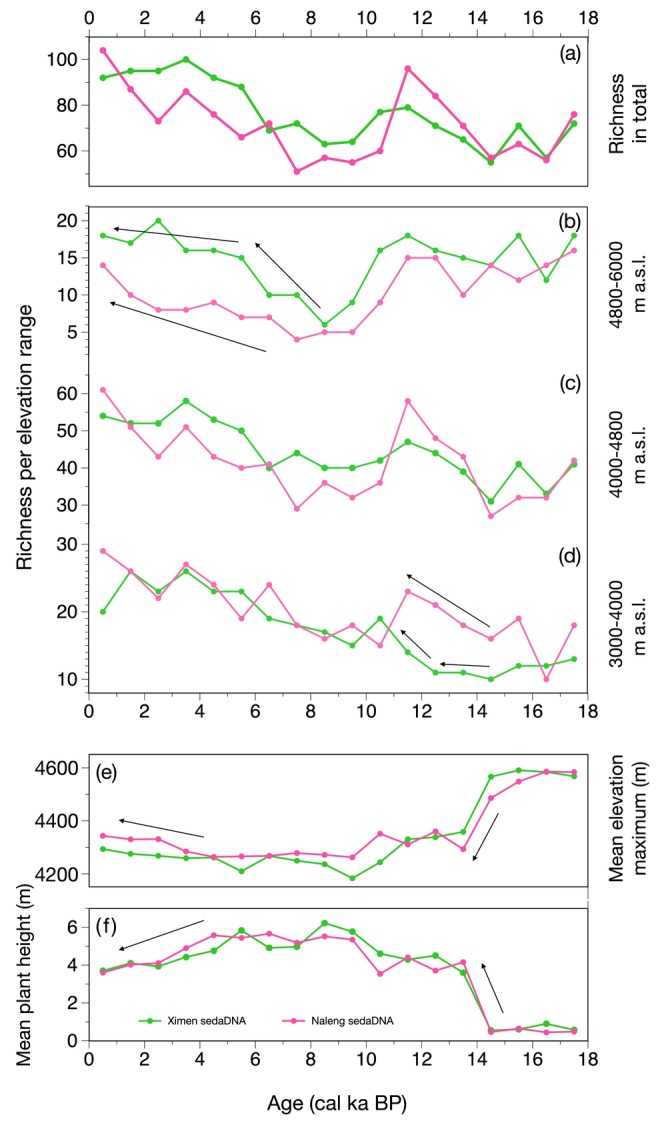
Comparison of long‐term richness and traits change between Lakes Ximen (green line) and Naleng (pink line). (a) Total plant richness. (b) Upland taxa assembly after the early‐Holocene warming was faster at Lake Ximen than at Lake Naleng. (c, d) Mid‐ and low‐elevation taxa show rapid richness increase after deglaciation at Lake Naleng due to good connectivity to refugia but a delayed pattern at Lake Ximen. (e) Mean taxa elevation maximum decreased strongly around 14 cal ka BP. (f) Mean plant height increased strongly after 14 cal ka BP and slightly decreased after 8 cal ka BP.

The LGM and late glacial (18–14 cal ka BP) plant metabarcoding records of Lakes Ximen and Naleng are dominated by taxa that currently occur in high‐alpine sparse vegetation including *Saxifraga*, *Meconopsis*, *Rhodiola*, *Pedicularis*, *Oxytropis*, *Ranunculus*, *Saussurea* subgen. *Eriocoryne*, *Kobresia*, *Polygonum*, *Carex*, and *Poa* (Figure 2). A strong vegetation turnover is inferred between 14 and 9 cal ka BP when many lowland taxa colonized the catchments including *Salix*, *Lonicera*, *Spiraea*, *Picea*, and *Betula*. While most trees and shrubs jointly colonized Lake Naleng around 14 cal ka BP, the colonization at Ximen occurred successively (Figures 2 and 3d). The early‐ to mid‐Holocene vegetation is characterized by high values for *Picea* and thermophilous shrubs like *Rhododendron*, *Spiraeae*, and *Lonicera*. After 8 cal ka BP, upland taxa start to increase in Lake Ximen and, slightly less, in Naleng. In the late Holocene, lowland woody taxa retreated from the catchment in Ximen, while those at Lake Naleng remained relatively stable. Further, high‐alpine taxa reappeared in both catchments, particularly at Lake Ximen (Figures 2 and 3b).

Long‐term richness changes in Lakes Ximen and Naleng show similar overall trends (Figure 3a). Lake Naleng shows rapid recolonization by lowland taxa (< 4000 m a.s.l.) after deglaciation compared with Lake Ximen (Figure 3d). The Lake Ximen record reveals an earlier late‐Holocene increase of alpine taxa (> 4800 m a.s.l.) compared with Lake Naleng (Figure 3b). There is a clear shift at 14 cal ka BP from high taxa elevation maxima and low mean plant heights at both sites (Figure 3e,f).

**FIGURE 4:**
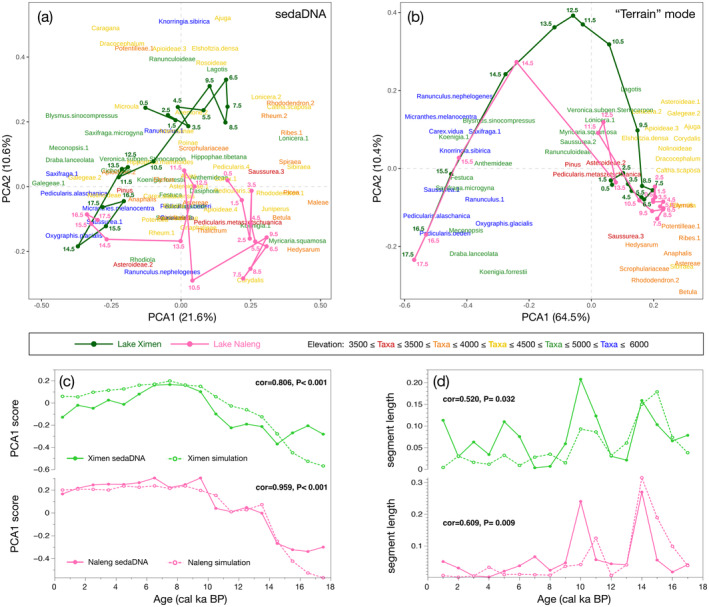
Biplots of ordination‐based trajectory analyses of sedaDNA proxy and species migration along river corridors (SMARC) “Terrain” mode simulation results. (a, b) Each line represents a trajectory, with time‐slice ages labeled (cal ka BP). The colors of the taxa labels are determined by their mean elevation maximum. Both biplots show a gradient from high‐elevation taxa on the left to low‐elevation taxa on the right. Comparison of sedaDNA proxy and “Terrain” mode simulation for (c) principal component analysis axis 1 (PCA1) scores and (d) segment lengths and showing Pearson's r of correlation analysis (cor) and associated p‐value. All results point to a similar community trend in proxy and model data.

These general patterns of compositional change are reflected by the results of the trajectory analyses using a joint PCA for Lake Ximen and Lake Naleng samples summarized on 1 ka time‐slices (Figure 4a,c). PCA axes 1 and 2 explain 21.6% and 10.6% of the variance, respectively. Both trajectories show a generally similar trend on PCA1 reflecting elevation gradients in the taxa distribution. Both trajectories start from low values during the glacial period, but while Lake Naleng PCA1 axis scores increase suddenly at 14 cal ka BP, Lake Ximen scores increase more gradually from 14 to 9 cal ka BP. Both trajectories reach their PCA1 maximum during the early and mid‐Holocene and show a reverse during the late Holocene. While PCA2 scores for glacial samples are similar to PCA1, the PCA2 scores for Lake Ximen emphasize the differences in the trajectories during warm periods, that is, the Bölling/Alleröd and early to mid‐Holocene. Interestingly, Lake Naleng PCA2 scores approach those of Lake Ximen during the Younger Dryas and late Holocene cold periods.

### 3.2 SMARC Simulation for Lake Ximen Compared to Lake Naleng

**FIGURE 5:**
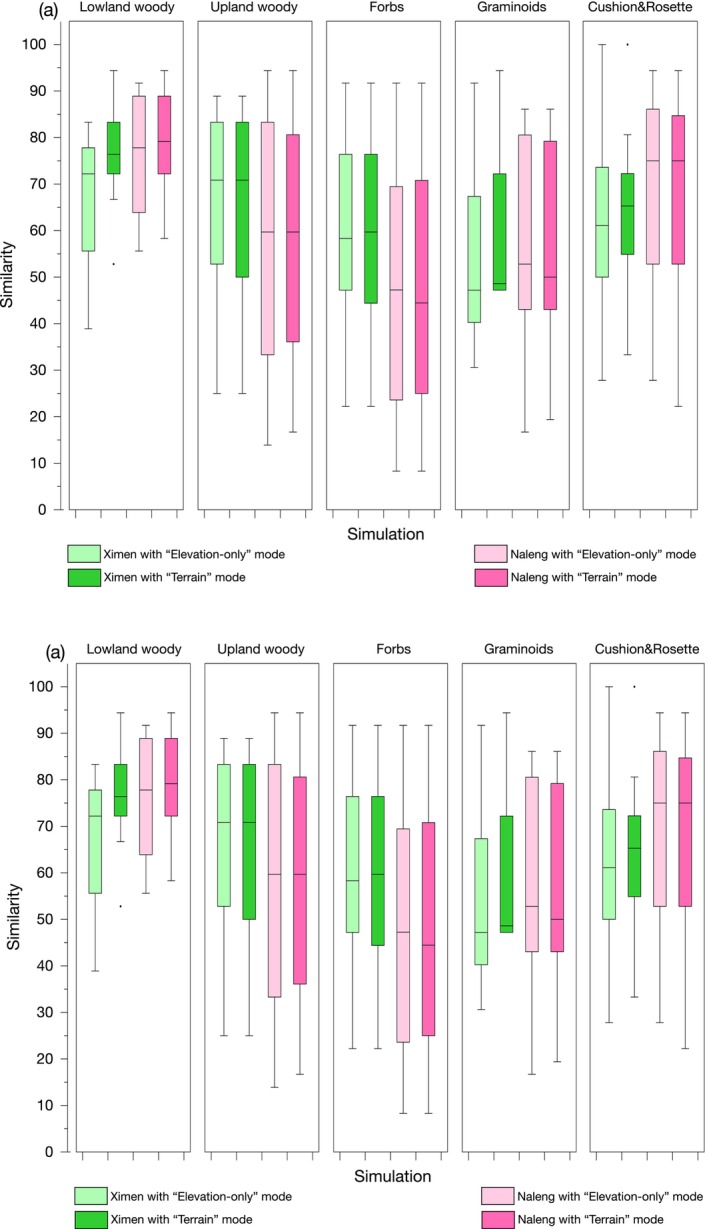
Comparison of species migration along river corridors (SMARC) model results of “Elevation‐only” mode and “Terrain” mode for certain growth forms. (a) Boxplot of similarity between SMARC simulation results and sedaDNA proxy. Compared to “Elevation‐only” mode (paler shading), the similarity improved with the “Terrain” mode only for Lake Ximen, particularly for lowland woody taxa, followed by cushion and rosette taxa. (b) Barplots of the percentage of taxa for which the proxy‐model agreement improved/worsened for “Terrain” mode and “Elevation‐only” mode. There is an improvement for most lowland woody taxa.

We ran the SMARC model for 148 comparisons in “Terrain” and “Elevation‐only” modes. There is a significant match between the “Terrain” simulation results and the sedaDNA data for 33.8% taxa, among which, 10 out of 16 woody taxa have significant correlations. Overall, the model‐proxy similarities are better for the “Terrain” simulations compared to “Elevation‐only” mode. This applies especially to lowland thermophilous woody taxa, that is, those that have an upper distribution elevation of < 4200 m, where 7 out of 8 taxa in Lake Ximen and 5 out of 8 taxa in Lake Naleng showed an improvement respectively, followed by cushion and rosette taxa. However, similarities did not improve for the upland woody taxa, that is, those that have upper elevation limits of > 4200 m, including Saliceae, *Dasiphora*, and *Hippophae* (Figure 5).

**FIGURE 6:**
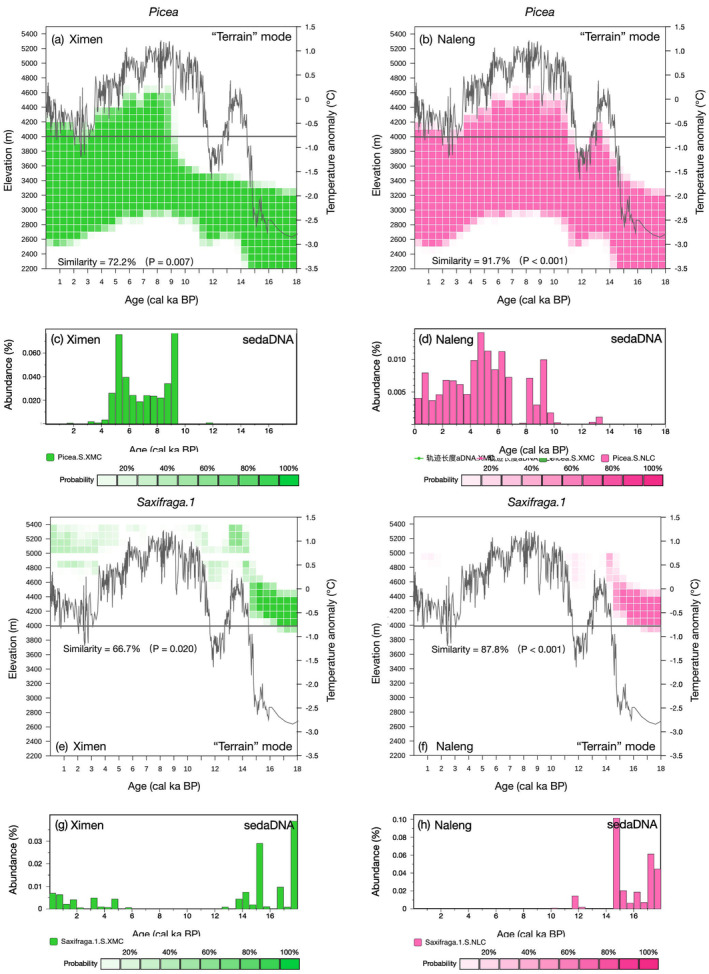
Elevation range changes of *Picea* and *Saxifraga* in species migration along river corridors (SMARC) model “Terrain” mode simulations (30 runs) since 18 cal ka BP compared to the relative abundance in sedaDNA proxy data for Lake Ximen and Lake Naleng. (a–d) *Picea*, a lowland woody taxa, shows a migration lag caused by poor connectivity to distant lowland due to the gentle downstream terrain at Lake Ximen. (e–h) Alpine *Saxifraga.1* reappeared earlier in Lake Ximen compared with Lake Naleng in the late Holocene due to good connectivity to steep upstream terrain.

Taking the lowland taxon *Picea* as an example, when the “Terrain” mode is used, the simulated colonization is much delayed at Lake Ximen due to the long distance to glacial lowland refugia but only slightly delayed at Lake Naleng because of the steep downstream terrain and much shorter distance to lowland (Figure 1c). Compared to the “Elevation‐only” mode, the similarity between simulation and sedaDNA proxy improved to 91.7% (*p* < 0.001) for Lake Naleng and to 72.2% (*p* = 0.007) for Lake Ximen in the “Terrain” mode (Figure 6a–d). For high alpine taxa, such as *Saxifraga*.1, the “Terrain” and “Elevation‐only” modes present similar results (Figure 6e–h, Appendix S1: Figure S1.2 e–h).

We used the simulations from all 126 taxa since 18 cal ka BP to set up compositional presence/absence time‐series datasets to implement trajectory analysis. The “Terrain” mode reproduced the peculiarities of the Ximen and Naleng trajectories (Figure 4b) but the “Elevation‐only” mode simulations did not (Appendix S1: Figure S1.1 b). For example, the trajectory analyses portray longer segments for Lake Naleng compared to Lake Ximen after 14 cal ka BP, which are seen in the “Terrain” mode but not so well in the “Elevation‐only” mode. Likewise, the Lake Ximen reversed trajectory reflecting the late Holocene cooling, which is not as obvious for Lake Naleng, is also better captured by the “Terrain” mode. This is confirmed by the correlation tests of the PCA1 scores (Ximen cor = 0.806, *p* < 0.001; Naleng cor = 0.959, *p* < 0.001) and trajectory segments (Ximen cor = 0.520, *p* = 0.032; Naleng cor = 0.609, *p* = 0.009) between the sedaDNA and the “Terrain” mode simulations (Figure 4c,d).

## 4 Discussion

### 4.1 Alpine Taxa in the Glacial Vegetation and Recolonization During the Late Holocene

SedaDNA and simulation results consistently indicate that taxa of sparse alpine vegetation, such as *Rhodiola*, *Meconopsis*, *Saxifraga*, *Saussurea* subgen. *Eriocoryne*, *Oxygraphis*, *Draba*, and *Galegeae*, dominated the catchments of both lakes Ximen and Naleng during the LGM until about 14 cal ka BP. This is in agreement with pollen records from the same cores (Kramer et al. 2010a; Herzschuh et al. 2014) and other pollen records from the eastern TP (Wischnewski et al. 2011). The sedaDNA record, in contrast to the pollen record, is not impacted by arboreal plant material originating from the lowlands and transported up‐slope by topographic winds (Herzschuh 2007). This confirms the local origin of the aDNA source (Pedersen et al. 2013).

A similar vegetation composition in the Lake Naleng and Lake Ximen catchments is indicated by the clustering of samples in ordination space (Figure 4a) and the consistency between the simulation and proxy evidence. Our high‐resolution taxonomic data reveal a widespread, homogenous Tibetan glacial flora whose present‐day distribution range is mainly in the upper alpine region. The inferred traits of the identified taxa of low mean plant height, high‐elevation habitat, and cushion and rosette growth forms (Figure 3e,f) further support a cold and dry climate during the glacial period (Herzschuh et al. 2023). We were able to retrieve many taxa from many samples using sedaDNA, which indicates that they occurred at both sites with sufficiently high biomass during the LGM. This is further evidence that populations were more widely distributed and had better connectivity than today. Compared to the present, this cold‐adapted flora was compositionally similar but richer; it does not represent a strongly impoverished relict flora in a restricted area. This aligns with the perspective that the TP was not covered by a massive ice shield but rather hosted several localized mountain glaciers (Shi, Zheng, and Li 1992).

The vegetation shows similarities with the Eurasian‐Beringian glacial vegetation (Willerslev et al. 2014; Courtin et al. 2021; Huang et al. 2021; Wang et al. 2021). Herbaceous plants were the dominant plant group with forbs more abundant than graminoids. The proportion of trees and shrubs is very low. Both forbs and graminoids are more species‐rich than previously considered with communities mainly composed of *Bistorta*, *Artemisia*, *Saxifraga*, *Pedicularis*, *Draba*, *Potentilla*, *Ranunculus*, *Salix*, *Caltha*, *Carex*, *Festuca*, *Poa*, and *Elymus*. This indicates that the vegetation was homogenous in the LGM, and some cold‐adapted species ranges are larger during glacials due to their spread to lowland areas (Stewart et al. 2010).

Our results also consistently indicate that some of these glacial flora taxa reappeared during the Late Holocene (Figures 2 and 4a) when the climate became colder and drier (Herzschuh et al. 2023). These cold‐resistant, xerophilous taxa often inhabit alpine gravel slopes and other specific patchy habitats (Xu, Li, and Sun 2014). Interestingly, they appeared earlier and with higher richness in Lake Ximen compared to Lake Naleng during the late‐Holocene cooling (Figure 3b). This points to a better connectivity to upland refugia for the Lake Ximen site. Lake Ximen's catchment has substantial nival and subglacial habitats which may have served as refugia for high‐alpine flora during the warm early and mid‐Holocene (Figure 1d). Such habitats are lacking in Lake Naleng's catchment, which made species more susceptible to extirpation. This implies that late Holocene re‐colonizations of the Lake Ximen site by alpine plants occurred randomly from other high alpine “islands” where populations survived.

This interpretation of the sedaDNA proxy is confirmed by the SMARC simulation (Figure 6e–h). Hence, our results support the proposed recurrent pattern of glacial expansion and interglacial contraction, known as the “sky island” system. Phylogeographic studies of glacial plant taxa have revealed highly random haplotypes with low genetic differentiation among populations but high genetic differentiation within populations (Luo et al. 2016). The slight late‐Holocene expansion has often been ignored in past phylogeographic studies (You et al. 2018). This may have obscured the global‐interglacial phylogenetic signal, further underlining the relevance of proxy time series.

### 4.2 Woody Taxa Migration out of Glacial Refugia in Uplands

Our sedaDNA proxy provides the first paleoecological evidence that several woody plant taxa had refugia on the TP during the LGM. This means that these taxa either survived the LGM directly in the catchments of the studied lakes, such as *Salix* or *Dasiphora*, or they quickly recolonized the catchments after 14 cal ka BP from nearby refugia, such as *Hippophae* and *Caragana*. These taxa are cold‐tolerant pioneer species that can survive in glacier‐ or permafrost‐impacted regions (Schlütz and Lehmkuhl 2009), and some of them can reproduce asexually in extremely harsh environments (Bhagwat and Willis 2008). This is reflected in our simulations where both the “Terrain” mode and “Elevation‐only” mode are almost indistinguishable in reproducing the proxy‐based species dynamics (Figure 5). This suggests that postglacial migration was not constrained by the complex downstream terrain, supporting the idea that these taxa either persisted in situ or in nearby upland glacial microrefugia, undergoing localized expansion during interglacial or post‐glacial periods. Also, phylogeographic studies of these taxa show that their upland populations have higher nucleotide and haplotypic diversities than those from the lowland edge of the TP and often have exclusive haplotypes on the TP platform (Wang et al. 2010; Ma et al. 2014), consistent with our inference of upland refugia.

### 4.3 Woody Taxa Migration out of Glacial Refugia at the Edge of the TP

Based on our sedaDNA and SMARC evidence we conclude that thermophilous woody taxa including *Picea*, *Betula*, *Sibiraea*, *Spiraea*, and some species of *Rhododendron* migrated from lowland refugia to the Ximen and Naleng catchments in response to postglacial warming. Our results are in line with molecular evidence from phylogeographic studies (Meng et al. 2007; Chen and Lou 2019; Khan et al. 2018; Fu et al. 2016). For these taxa, higher haplotype diversity is observed in the lowland refugial populations at the eastern edge of the TP compared with the upland populations, pointing to a bottleneck and founder effect when colonizing upland habitats (Gugerli et al. 2001).

The proxy data show that the upland was colonized between 13 and 10 cal ka BP, which is considerably later than the rapid glacier melt at around 14 cal ka BP documented in the Naleng and Ximen lake sediments. This suggests a lag in vegetation response to climate, indicating a disequilibrium between climate and vegetation. This disequilibrium led to the differential response to the Younger Dryas event, which was stronger for Naleng where most warm‐loving taxa had already colonized but disappeared again when the climate cooled, becoming again more similar to that around Lake Ximen (Figure 4a).

Our interpretation of colonization being constrained by distance to the lowland refugia is confirmed by the simulations. For example, the similarity for woody taxa with upper elevations of < 4200 m was better for results from the “Terrain” mode compared with the “Elevation‐only” mode (Figure 5). This applies not only to individual taxa but is of importance at the community level. The trajectory segment lengths of the proxy data and the “Terrain” mode simulation results show a much better fit than for the “Elevation‐only” mode simulation (Figure 4b,d, Appendix S1: Figure S1.1 b,d). Hence consideration of the mountain terrain—in our case, the site‐specific migration route along the rivers—represents a first‐order factor for the postglacial vegetation trajectory on the TP and emphasizes the complex relationship between temperature and species assembly.

The terrain downstream of Lake Ximen is relatively gentle, while downstream of Lake Naleng is steeper. The Hengduan Mountains river valleys are characterized by nutrient‐rich soil that likely served as important natural corridors for the migration of plant species (Rana et al. 2020; Holeštová and Douda 2022). The upper treeline may have been as low as 3350 m a.s.l. in the eastern TP during the LGM (Frenzel, Bräuning, and Adamczyk 2003). The distance to each study site from a corresponding location at this elevation via the river valley is only 100 km for Naleng while it is 400 km for Ximen (Figure 1). Additionally, the downstream area of Naleng is humid, while the downstream area of Ximen is more arid (and was likely even more arid during the glacial period) (Qin, Zhao, and Cao 2022).

We find that the woody taxa *Lonicera*, *Spiraea*, *Sibiraea*, *Rhododendron*, *Picea*, and *Betula* almost synchronously colonized the catchment of Naleng at 13.3–13.0 cal ka BP. In contrast, they successively colonized the catchment of Lake Ximen between 13.5 and 9.0 cal ka BP (Figure 2). For example, *Rhododendron* appeared at 13.5 cal ka BP at Lake Naleng while it reached Lake Ximen as late as 9.2 cal ka BP. Likewise, *Picea* colonization of Lake Ximen lags behind Lake Naleng. Also, there is no obvious increase of *Betula* in the northeastern TP between 14 and 11 cal ka BP (Zhou et al. 2010; Herzschuh et al. 2014). The sedaDNA colonization signal at 9.9 cal ka BP of Ximen lags behind the pollen‐based regional colonization signal by more than a thousand years (Herzschuh et al. 2014). In contrast, there is almost no lag observed for Lake Naleng (13.2 cal ka BP), which shows concomitant results for both sedaDNA and pollen (Kramer et al. 2010a; Liu et al. 2021). Hence, for these taxa, our results support the “expansion” hypothesis (Meng et al. 2007), that is, platform populations are derived from a common colonization event from the edge of the TP. This is in agreement with phylogeographic studies, which have inferred a marked founder effect (Gugerli et al. 2001).

### 4.4 General Conclusions About Connectivity in Mountain Terrain and Future Changes

Our results indicate that connectivity to refugia is a first‐order factor for species migration in addition to elevation‐related species' climatic niches shaping the postglacial vegetation trajectory in mountainous terrain. In our study, connectivity refers to a property of landscapes, that encompasses the degree to which the landscape facilitates or impedes individuals' flows across space among resource patches (Baguette et al. 2013). This has hitherto largely been ignored when predicting mountain vegetation responses to climate change and related risk assessment.

Our modeling study indicates that dispersal‐limitation may represent the key process causing migrational lags although we did not systematically assess other processes. Dispersal‐limitation implies that not all suitable habitat patches will be occupied by species with corresponding niche properties (Ehrlén and Eriksson 2000) despite occurring in the regional species pools (Ozinga et al. 2005). Our study is among the first that highlights the potential influence of terrain on dispersal‐related migrational lags from consistent proxy and simulation time series.

Complex mountain terrain results in uneven vegetation trajectories between sites with similar climate conditions and climate history, mainly because of differences in the connectivity to refugia. Our results indicate that even a single site can show uneven responsiveness to a warm or cold climate depending on the shape of its upstream and downstream relief. We could show that the steep terrain downstream of Lake Naleng enhances its connectivity to glacial lowland refugia. In contrast, gentle terrain over long distances, such as downstream of Lake Ximen, makes for weak connectivity to lowland refugia. For ongoing changes, this implies that colonization of mountain areas by lowland taxa will be uneven, even at a regional scale. Strongly disconnected regions such as the northeastern TP will be reached much later compared to regions with steep terrain on the southern TP.

Likewise, terrain differences among our sites defined the different connectivity to upland refugia in the past. Lake Ximen has direct connectivity to high‐alpine habitats and was therefore quickly recolonized during late‐Holocene cooling. In contrast, the limited upper elevation maximum in the Lake Naleng catchment restricted recolonization. High‐alpine taxa have island‐like distribution ranges due to mountain topography making it difficult for them to migrate to new habitats (Jump and Peñuelas 2005). In analogy to early‐to‐mid Holocene warming, alpine plants will be at risk of local mountain‐top extinction, which will become more likely when future warming surpasses past thresholds. Relocation of subnival taxa to colder and more suitable habitats (Liang et al. 2018) making use of alpine plant seed banks (Breman et al. 2021) may represent a last measure to conserve the rich Tibetan upland flora in natural habitats.

As sedaDNA analyses are time‐consuming, only few sites have been investigate so far. However, despite this study is based on data from only two lakes on the eastern TP, our proxy data and simulations consistently show that not only are rare species affected by lagged migration but also the entire plant community. Poor connectivity to regional species pools results in complex site‐specific plant assemblies and vegetation trajectories and ultimately vegetation‐climate disequilibrium (Svenning, Normand, and Skov 2008). The migration lag of lowland taxa is a major factor in vegetation‐climate millennial‐scale disequilibrium (Svenning, Normand, and Skov 2008). This might be the reason for the observed strong vegetation lags in remote areas such as Chukotka (Herzschuh et al. 2016) or northern Eurasia (Svenning and Skov 2004).

## Author Contributions

**Wei Shen:** conceptualization (equal), data curation (lead), formal analysis (lead), funding acquisition (equal), investigation (lead), methodology (equal), software (equal), validation (equal), visualization (lead), writing – original draft (lead), writing – review and editing (lead). **Stefan Kruse:** formal analysis (equal), funding acquisition (equal), methodology (equal), software (lead), supervision (equal), validation (equal), writing – original draft (equal), writing – review and editing (equal). **Sisi Liu:** writing – review and editing (equal). **Kathleen Stoof‐Leichsenring:** conceptualization (equal), data curation (equal), project administration (equal), resources (equal), supervision (equal), validation (equal), writing – review and editing (equal). **Ingolf Kühn:** validation (equal), writing – review and editing (equal). **Wenjia Li:** visualization (supporting), writing – review and editing (lead). **Xianyong Cao:** writing – review and editing (equal). **Zhi‐Rong Zhang:** resources (equal), writing – review and editing (supporting). **Chun‐Xia Zeng:** resources (equal), writing – review and editing (supporting). **Jun‐Bo Yang:** funding acquisition (supporting), resources (equal), writing – review and editing (equal). **De‐Zhu Li:** funding acquisition (supporting), resources (equal), writing – review and editing (equal). **Ulrike Herzschuh:** conceptualization (lead), data curation (lead), formal analysis (lead), funding acquisition (lead), investigation (lead), methodology (lead), project administration (lead), resources (lead), software (lead), supervision (lead), validation (lead), visualization (lead), writing – original draft (lead), writing – review and editing (lead).

## Conflicts of Interest

The authors declare no conflicts of interest.

## Supporting information


Appendix S1.

**Appendix S2.** Tables of the raw and processed data used to generate the figures.

## Data Availability

The raw NGS sequencing data that support the findings of this study have been archived in the European Nucleotide Archive (ENA) with the accession code: PRJEB72448. The data processing process of the OBITools pipeline and merging, denoising, and rarefaction was stored at Zenodo: https://doi.org/10.5281/zenodo.10794022. SMARC Model v1.0 is available on Zenodo: https://doi.org/10.5281/zenodo.10732098. The filtered sedaDNA datasets and statistical analyses during this study are provided in Supporting Information.
