# Supplementary material for: Post‐Glacial Vegetation Trajectories on the Eastern Tibetan Plateau Reflect Millennial‐Scale Migration Lags in Complex Mountain Terrain Based on Sedimentary Ancient DNA and Dynamic Dispersal Modeling
Source: Ecol Evol. 2025 Jan 22;15(1):e70862. doi: 10.1002/ece3.70862 (PMC11754073; doi:10.1002/ece3.70862)
Supplement: Supplementary file 1 — Appendix S1. [file ECE3-15-e70862-s002.docx]

# **Post-glacial vegetation trajectories on the eastern Tibetan Plateau reflect millennial-scale migration lags in complex mountain terrain based on sedimentary ancient DNA and dynamic dispersal modeling**

**Supplementary material**

Table of contents

S1. Results of Elevation-only Model

S2: ODD protocol for SMARC Model

S3. Parameterization and sensitivity analyses

S4. Workflow of SMARC Model

#

# **S1. Results of “Elevation-only” mode**
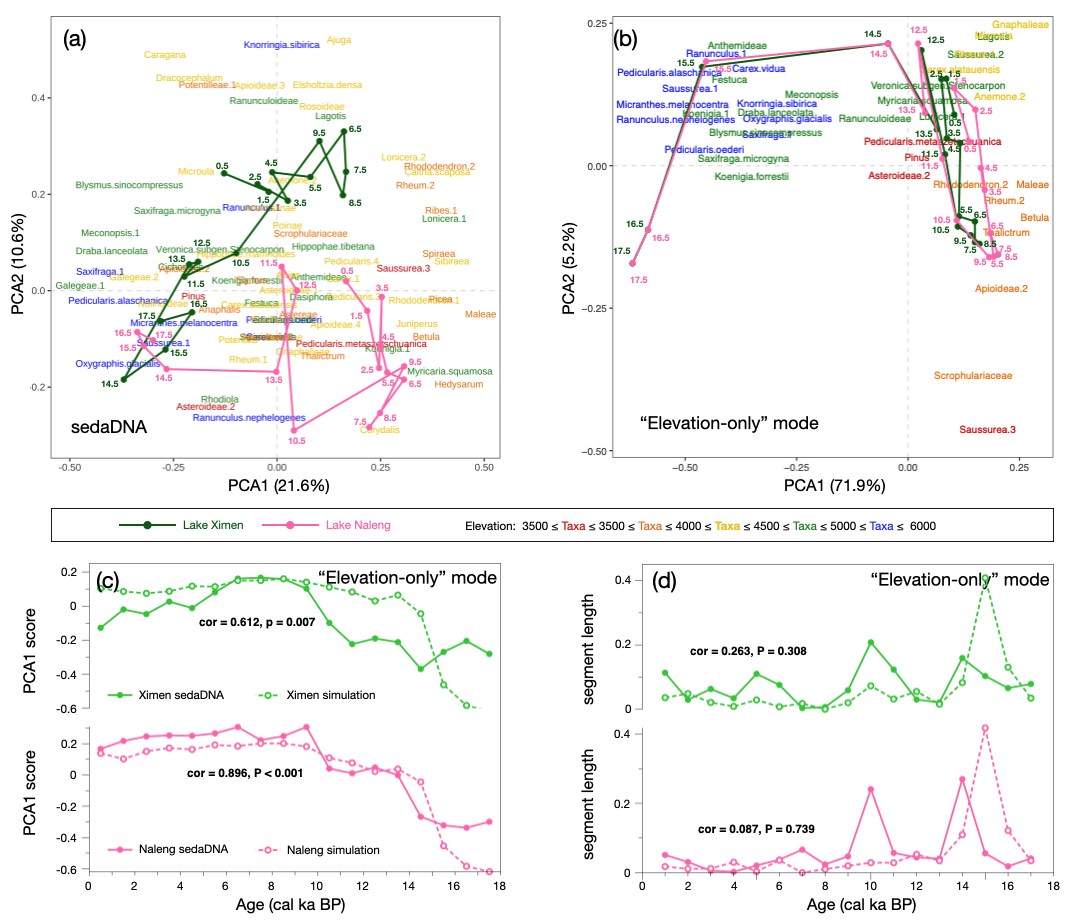


**Figure S1.1**

Biplots of trajectory analyses (a,b), line plots of PCA1 scores (c), and segment lengths (d) for sedaDNA and "Elevation-only" mode simulations. Lines represent trajectories, while the points on the lines indicate age (cal ka BP). The color of the taxa labels reflects their mean elevation maximum. There is a gradient from high-elevation taxa on the left to low-elevation taxa on the right. We used the simulations of all 126 taxa to set up compositional presence/absence time-series dataset, presented in the trajectories of “Elevation-only” mode and sedaDNA proxy (Fig. S1.1a,b). Only the general trend of the PCA trajectories of “Elevation-only” mode is consistent with the PCA results from sedaDNA data presence/absence data, with a shift from cold glacial to warm deglaciation. This is confirmed by Pearson correlation analysis of the PCA1 axis (Ximen cor=0.612 p=0.007, Naleng cor=0.896 p<0.001) (Fig. S1.1 d). However, PCA results from the “Elevation-only” mode show similar trajectories for Ximen and Naleng and a similar response to climate warm/cold events. This is inconsistent with the different colonization patterns and degree of response to climatic events between these two sites indicated by the sedaDNA proxy. This is also confirmed by the low correlation for the segment lengths between sedaDNA and simulation data (Ximen cor=0.263 p=0.308, Naleng cor=0.087 p=0.739) (Fig. S1.1 d).


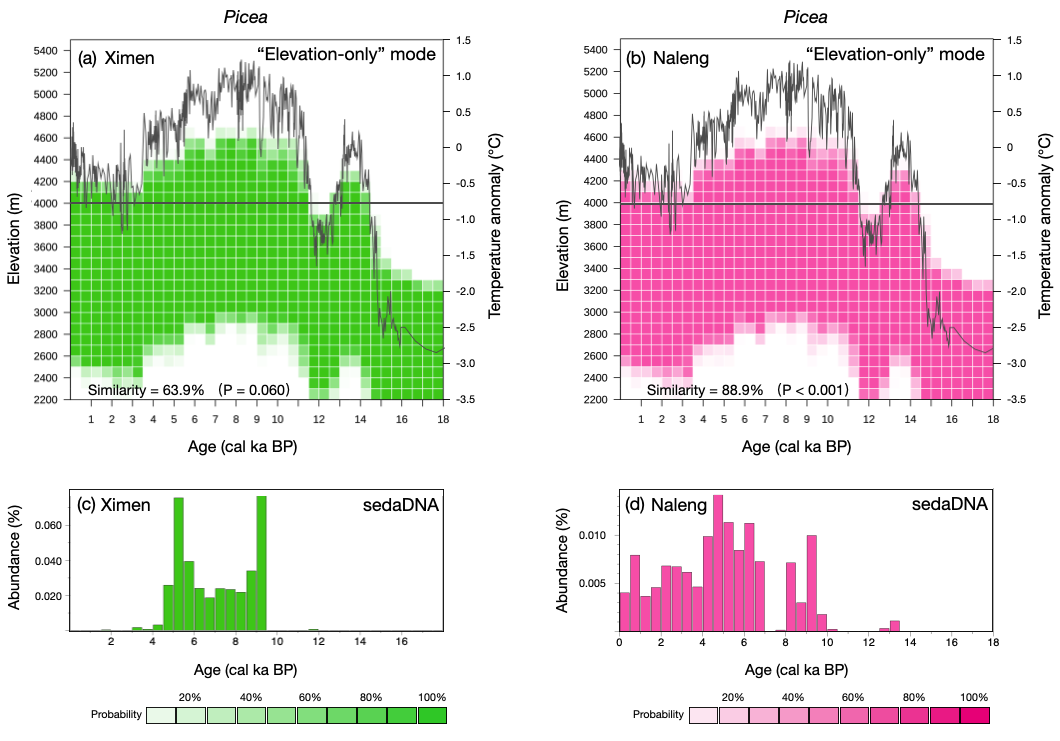


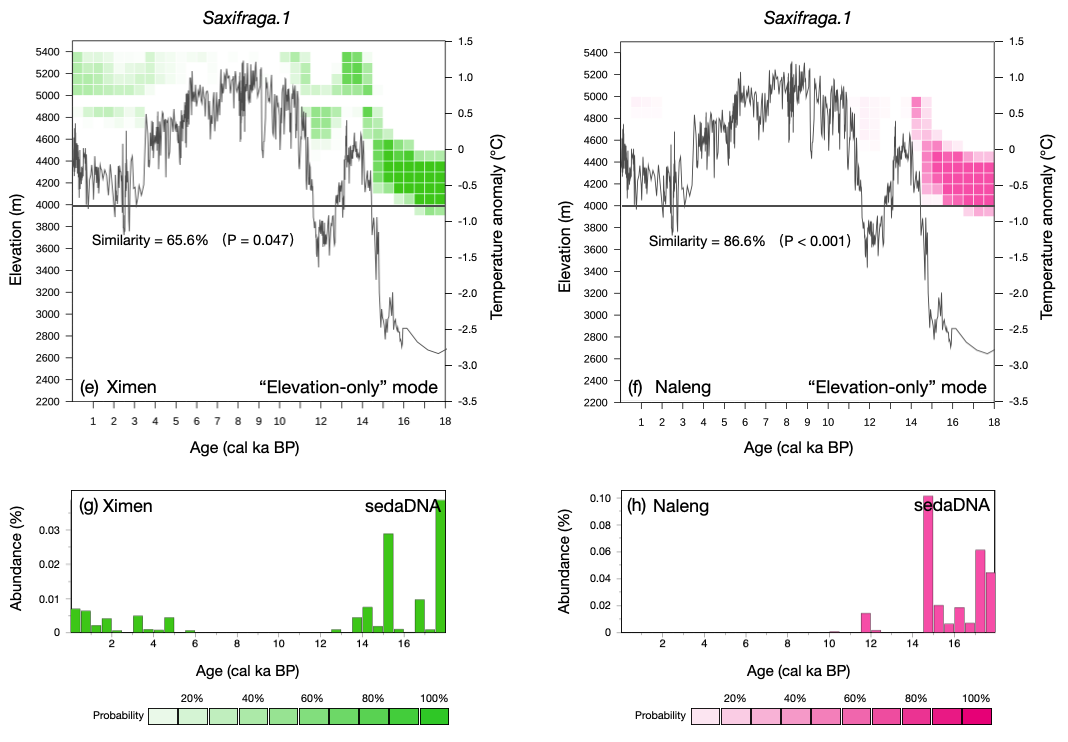


**Figure S1.2**

Comparison of “Elevation-only” mode simulations (based on 30 repeats) for elevation distribution range changes of specific taxa since 18 cal ka BP with the abundance of sedaDNA proxy. The probability of a taxon occurring in space is represented in grayscale. For example, the simulation of *Picea* using the “Elevation-only” mode driven by temperature change yields similar results for Lake Ximen and Lake Naleng because of similar modern temperatures. The simulated *Picea* presence has an 88.9% similarity (P<0.001) compared to the sedaDNA data from Naleng but only a 63.9% similarity (P=0.060) with Lake Ximen (Fig. S1.2 a, b). For alpine taxa, with "Elevation-only" mode, the upper alpine habitats limitation is different. So the simulation results of the two study areas are different. The simulated *Saxifraga.1* presence has an 86.6% similarity (P<0.001) compared to the sedaDNA data from Naleng but only a 65.6% similarity (P=0.047) with Lake Ximen (Fig. S1.2 a, b).

# **S2: ODD protocol for SMARC model**

The model description follows the updated ODD (Overview, Design concepts, Details) protocol (Grimm et al. 2006, 2010).

| **Category** | **Element** |
| --- | --- |
| Overview | **1. Purpose**  The dynamic dispersal model SMARC was set-up to simulate the colonization and the dynamics of plants in a lake catchment. It serves as a tool to test the impact of dispersal lags (Svenning et al. 2008) stemming from complex, mountainous terrain along river corridors on post-glacial vegetation trajectories and thus on the observable plant biodiversity.  We aimed for a simple model requiring limited computational resources which could still be directly compared to plant proxy data from lake sediments with respect to spatial, temporal, and taxonomic resolution. |
|  | **2. Entities, state variables, and scales**  The model consists of two hierarchical levels characterized by a few parameters and state variables: (1) spatial units, that is, the ***terrain*** that consists of positions that can be colonized and (2) agents that are the individual ***plant taxa*** (see Table S2.1 and Table S2.2).    The ***terrain*** is the simulation area and is a linearized transect following the natural path of a river. We used two different rivers that pass through Lake Naleng and Lake Ximen, with a length of 900 and 570 m, respectively. Each terrain has a certain step size, here 250 m, and each plant taxon is either present or absent at the positions in a given time step. We consider only those ***taxa*** shared by the two lakes Ximen and Naleng for modeling to enable a comparison of differences. Each of the 126 taxa sequences corresponds to one or more species and we calculated mean elevation values based on the known distribution ranges.    Simulation runs proceed in yearly time steps. We performed simulations for years 0–50,000 yr BP during which, for the last 16,000 yr BP, high-resolution (=20-yr steps) temperature proxy series were available. This was prolonged by using a reconstructed temperature anomaly back to 22,000 yr BP. Additionally, to reach stabilization of establishment of taxa in the terrain, simulations were preceded by a stabilization period of 28,500 years and before 18,000 yr BP the probability of establishment (pcolonise) is doubled. All simulations start from bare ground and in each time step taxa can colonize new positions in the terrain if suitable positions are available even after a taxon went extinct.    A local sensitivity analysis (+/-5% change in parameter values) was performed to assess the impact of the parameterization on simulation results.  Table S2.1. Parameters in the model SMARC   \|  \| **Abbreviation** \| **Value** \| **Dimension** \| \| --- \| --- \| --- \| --- \| \| *Terrain* \| \| \| \| \| *Temperature lapse rate* \| Templapse \| 0.55 \| °C/100 m elevation \| \| *Lateral step size to extract the elevation input data along the river corridors* \| EleStep \| 250 \| m \| \| *Time step, used to generate environment from temperature input data with a 20-year resolution by linear interpolation with* \| TimeStep \| 25 \| yrs \| \| *Lake elevation* \| LakeEle \| 4000 \| m a.s.l. \| \| *Plant taxon* \| \| \| \| \| *Factor moderating the colonization probability of a position* \| distpot \| 3 \| dimensionless scaling factor \| \| *Probability a taxon enters the simulation area* \| pinitial \| 80 \| % \| \| *Probability a taxon colonizes a position grid cell* \| pcolonise \| 0.5 \| % \| \| *Maximum dispersal distance per year* \| dispdist \| 100 \| m/year \|     Table S2.2. State variables of the structures in the SMARC model   \|  \| **Abbreviation** \| **Value** \| **Dimension** \| \| --- \| --- \| --- \| --- \| \| *Terrain* \| \| \| \| \| *Time step* \| t \| {0…50000} \| yrs BP \| \| *Elevation of the position taken initially from DEM input data and modified for each time step according to temperature lapse rate* \| TerrainReshape_x,t \| variable \| m a.s.l. \| \| *Position along the river corridor starting at lowest level (2200 m a.s.l. )* \| x \| variable \| m a.s.l. \| \| *Presence/absence of a certain taxon* \| - \| {0\|1} \| dimensionless \| \| *Plant taxon* \| \| \| \| \| *Median distributional breadth, calculated by the absolute range divided by the median of observations* \| DistriBreadth \| variable \| m a.s.l. \| \| *Minimum elevation of modern presence of species that are aggregated into the plant taxon* \| MinEle \| variable \| m a.s.l. \| \| *Maximum elevation of modern presence of species that are aggregated into the plant taxon* \| MaxEle \| variable \| m a.s.l. \| \|  \|  \|  \|  \| |
|  | **3. Process overview and scheduling**  The program code is implemented in R version 4.1.0 (R Core Team 2021) and simulations separately run for each considered taxon and river terrain. In each initialization phase of each simulation run, the terrain data are loaded. It proceeds in 25-year time steps from the beginning to the end of the compiled temperature series.    SMARC has the following sequence for each time step: (1) Calculate the potential ecological niche distribution of the taxa at that time based on current presence observation and digital elevation model combined with the temperature lapse rate and the temperature input data, (2) simulate taxa colonization and retreat. When a taxon is not yet present it can establish in the terrain with a certain probability and colonizes the lowest potential position. Without seed dispersal limitation all potential cells can be colonized with a certain probability. Alternatively, a dispersal limitation can be set, ignoring complex diffusion patterns and differences among species for simplification. When a taxon has colonized an area, the probability of reaching novel positions depends on its median distributional breadth. Further, the colonization probability is moderated by assuming that dispersal ability increases with the number of currently colonized positions. If it advances, it reaches at least one step in elevation towards the maximum distance. The same procedure applies for retreating to lower elevations. Extinction in cases where environmental conditions are outside the species’ niche, is harsh at both edges. |
| Design concepts | **4. Design concepts**  **• Basic principles**  The model serves as a tool to test the impact of dispersal lags (Svenning et al. 2008) stemming from complex, mountainous terrain along river corridors on post-glacial vegetation trajectories and thus on the observable biodiversity (Svenning and Sandel 2013, Snell and Cowling 2015, Tiebel et al. 2020).  In the mountainous alpine areas, climate is a strong determinant of the potential niche for plant species. We used elevation as a predictor variable and computed the niche for a taxon at a certain time step by applying a temperature lapse rate and constraining its colonization by seed dispersal over a specific terrain. We used here only one driver, the temperature anomaly, and the choice was made based on its importance in mountainous alpine areas (Went 1953) and to have a minimally complex model.  We aimed for a simple model requiring limited computational resources which could still be directly compared to plant proxy data from lake sediments with respect to spatial, temporal, and taxonomic resolution. The model was built following pattern-oriented modeling (Grimm and Railsback 2005) and based on a pure model in which taxa can colonize all potential grid cells falling in their niche range. Probability and dispersal-limitation were included to match observed trajectories of taxa. Parameters were based initially on literature values or educated guesses and fitted so that the model could reproduce observed patterns.    The SMARC model is based on the following fundamental assumptions:  • The terrain (elevation) along the river corridor remained unchanged.  • Observed species are at (pseudo-)equilibrium with their environment.  • Relevant ecological drivers (or proxies) of species distributions are included.  • Species exhibit genetic conservatism, maintaining relatively stable ecological niches.  • The species aggregated into the taxa groups have a similar niche.  • Interspecies interactions are ignored.  • Sampling of observation data is adequate and representative.    **• Emergence**  Biodiversity, expressed as the number of taxa present in the lake catchment at a certain time step, can be different for similar dispersal lags modulated by the shape of the terrain (steep versus moderate slopes).    **• Adaptation**  The colonization probability is moderated by assuming that dispersal ability increases with the number of currently colonized positions (qualitatively for >1 and <=10 positions, probability is doubled and for >10 positions it becomes three times more likely).  **• Objectives**  The increased success of colonizing new positions when a larger number is colonized assumes that the number of seed dispersal events increases and hence together with more long-distance seed dispersal events faster colonization rates can be met.    **• Learning**  No other learning behavior is included other than the increase of dispersal ability as laid out above.    **• Prediction**  Not applicable.    **• Sensing**  An individual plant taxon can sense its environment by the terrain position and the current-state variable of elevation. In the range of the dispersal maximum, it either colonizes new positions or goes extinct if environmental conditions fall outside the taxon’s niche.    **• Interaction**  No interactions between the taxa are considered. Information is shared within a taxon via the terrain positions that can either be colonized or empty.    **• Stochasticity**  To allow for variability and to model the stochastic nature of establishment processes, the models draw random numbers (RNG version Mersenne-Twister in r-function runif from base stats package) for tests, for the chance of entering the terrain when environmental conditions allow establishment (pinitial), as well as the establishment probability (pcolonise).    **• Collectives**  Not applicable.    **• Observation**  In each simulation step, the presence or absence of each taxon in the catchment of the lake is computed. If the upper limit of the simulated distribution range of a considered taxon is above the lake elevation, we use a 1 to represent if it appears in the catchment and 0 if it is absent.  Finally, if a taxon is present in 80% of the repeated simulations (N=30) it is retained for final analysis. The output data are merged into 500-year time slices for comparability. |
|  | **5. Initialization**  The model starts with reading the fixed parameters and reading input files, which are temperature anomaly, list of taxa, and the modern terrain along the river corridors. Each simulation starts for each taxon with an empty terrain at 50,000 yr BP. |
|  | **6. Input data**  To provide the necessary input data for each ***taxon*** on the level of proxy data, elevation distribution data for plant species potentially occurring in the region of the lakes were collected from the database of Flora of China (www.iplant.cn, Accession date March 8th 2022). In total, 529 species were aggregated to 126 plant taxa found with >0.03% sequence abundance in sedimentary ancient DNA datasets of the two lakes (with median=2, mean=4, min=1, max=29 species per taxon). The taxonomic reference system follows NCBI (Schoch et al. 2020).  ***Terrain*** profiles along the rivers of both Ximen and Naleng, located on the eastern Tibetan Plateau, China, were extracted from the 90-m resolution SRTM digital elevation model (Jarvis et al. 2008).  **For temperature forcing**, we matched the reconstructed (based on δ^18^O) high-resolution and variable temperature record from Dongge Cave to fit with the coarsely reconstructed temperature anomaly curve for the Northern Hemisphere (30°–90°N) since the last deglaciation (Fig. S2.1) (Dykoski et al. 2005, Shakun et al. 2012, Kruse et al. 2021). To allow the taxa to reach equilibrium with the climate data at the beginning of the series, the temperature anomaly for 21.5 cal ka BP was applied to all years back to 50 cal ka BP.    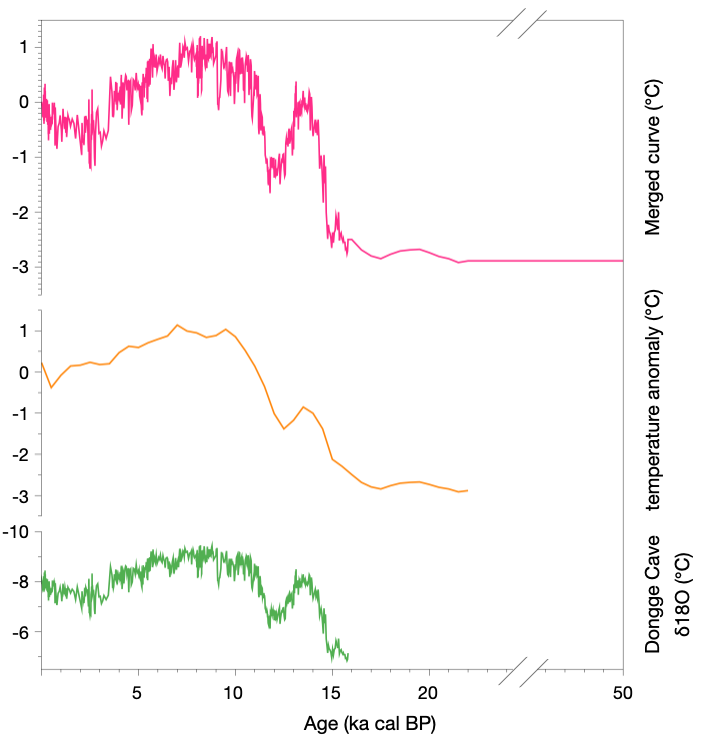  Figure S2.1. Climate synthesized record  There are measurement errors and biases in the input data: SRTM has a minimum vertical accuracy of 16 m absolute error at 90% confidence (root mean squared error (RMSE) of 9.73 m) worldwide (Mukul et al. 2017); temperature: 0.16‰ (mostly below 0.10‰) (16,000 BP–present) (Dykoski et al. 2005) and below 0.18% (16,000–22,000 BP) (Shakun et al. 2012, Kruse et al. 2021). |
|  | **7. Submodels**  7.1 The potential ecological niche distribution of the taxa at any time is calculated by applying a temperature lapse rate of 0.55 °C*100 m^-1^ (Li et al. 2013) to the temperature anomaly.  7.2 Taxa colonization and retreat along the terrain is estimated. When a taxon is not yet present it can establish in the terrain with a probability of 80% and colonizes the lowest potential position with a 1% chance. Without seed dispersal-limitation in the “Elevation-only” mode, the potential cells can be colonized with a chance of 80%. Alternatively, in the “Terrain” mode, a dispersal-limitation can be set to 100 m yr^-1^ (Svenning and Sandel 2013, Snell and Cowling 2015, Tiebel et al. 2020), ignoring complex diffusion patterns and differences among species, for simplification. When a taxon has colonized an area, the probability of reaching novel positions depends on its median distributional breadth (calculated by the absolute range divided by the median of observations). The colonization probability is moderated by assuming that dispersal ability increases with the number of currently colonized positions (qualitatively for >1 and <=10 positions, probability is doubled and for >10 positions it becomes three times more likely). If it advances it reaches at least one step in elevation towards the maximum distance. The same procedure applies for retreating to lower elevations. Extinction where environmental conditions are outside the taxon’s niche is harsh at both edges. |

#

#

# **S3. Parameterization and sensitivity analyses**

To explore the robustness of the model results to changes in the parameters and to reveal parameters the simulation is sensitive to, we performed a local sensitivity analysis. The model parameters were first set to match visually the observation of one alpine taxon (*Saxifraga.1*) and one taxon with a contrasting and low-elevation distribution range (*Picea*). We tested changing all parameters in the model by +/- 5% of the value (Table S3.1) and compared the simulation results to the observation from sedaDNA data. We transformed sedaDNA data from abundance data to present/absent (binary) data. Then for each time step, we evaluated from the model results (taxon distribution range) whether the taxon occurred above 4000 m a.s.l. or not. If yes, we recorded it as a 1, which means it is in the catchment, otherwise we recorded it as a 0, which means the taxa retreated or became extinct in the catchment. From this, we made sure that the simulation data of presence/absence of a taxon is in a format similar to the sedaDNA proxy data. A simple matching coefficient is introduced to measure the similarity between the sedaDNA and simulation data. To test the significance of the similarity between two binary vectors, the default 999 permutation test for the p-value of the simple matching coefficient (SMC) was implemented.

For each parameter combination, we conducted 5 independent simulations for each taxon at each site. Each run generated a SMC and a permutation test. The average of the similarity results from five runs of the reference combination serve as the reference benchmark. Then, we changed the parameters according to Table S3.1, generating results from five runs. The sensitivity is calculated using the function ((S ± 5%) - Sref) / Sref.

# **Results of the parameter tests**

Looking at the similarity value alone, a low value of 0.64 ± 0.06 similarity ( -9.5% ~ 12%) shows that taxa in the Ximen catchment are more sensitive than the taxa in Naleng (Fig. S3.1). This observation can be seen in the sensitivity analysis as well. The results of the sensitivity analysis show that the simulations are relatively robust to parameter changes for mid-to-lowland taxa, which exist in a relatively wide range of niches, and did not go extinct in the regional environments (Fig. S3.2a,b, Fig. S3.3a). For the mid-to-lowland taxon (*Picea*), changing the parameter pcolonise had the strongest effect on the model results. The others had approximately 3% change, meaning that allowing larger dispersal distances would lead to more area covered and would improve the model fit. However, the results for the alpine taxa are more sensitive with a median of ~16% sensitivity for the parameter pcolonise as well. Hence the simulation results do not strongly depend on the choice of the maximum dispersal distance.

Table S3.1. Model parameters and tested ranges in the local sensitivity analysis.

| Description | Abbreviation | Dimension | Reference value | Forward fine-tuning | Backward fine-tuning |
| --- | --- | --- | --- | --- | --- |
| Maximum dispersal distance per time step | dispdist | m/year | 100 | 105 (+5%) | 95 (-5%) |
| Probability a taxon enters the simulation area | pinitial | - | 0.80 | 0.84 (+5%) | 0.76 (-5%) |
| Probability a taxon colonizes a position | pcolonise | - | 0.005 | 0.00525 (+5%) | 0.00475 (-5%) |
| Factor moderating the colonization probability of a position | distpot | - | 3 | 3.15 (+5%) | 2.85 (-5%) |


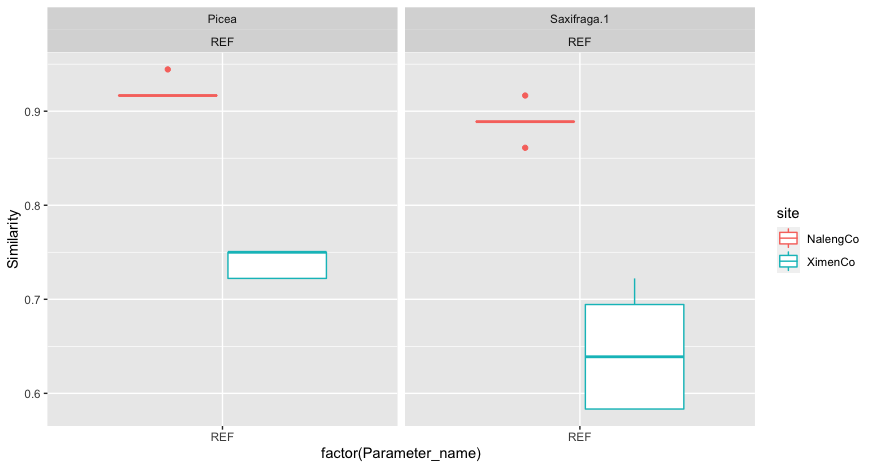


Figure S3.1. Similarity of observed versus simulated plant taxon (*Picea* low elevation and *Saxifraga.1* high alpine) presence in the catchment of the two lakes Naleng and Ximen using the standard parameters of the model (see Table S3.1).


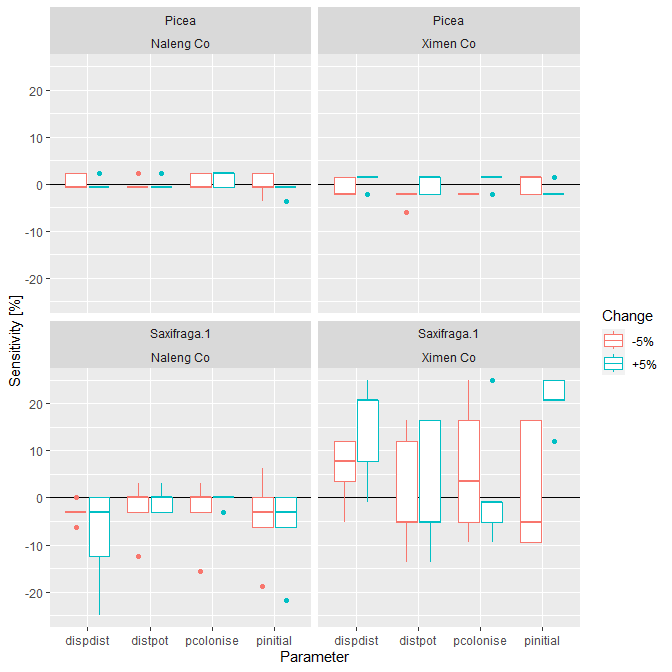


Figure S3.2. Sensitivity of the model outcome (expressed as similarity of plant taxa present in the lake catchment) to +/- 5% changes in the model parameters (for details on each parameter see Table S3.1). The sensitivity values are given for two taxa (rows: *Picea* low elevation and *Saxifraga*.1 high alpine) and two lakes (columns: Naleng and Ximen).


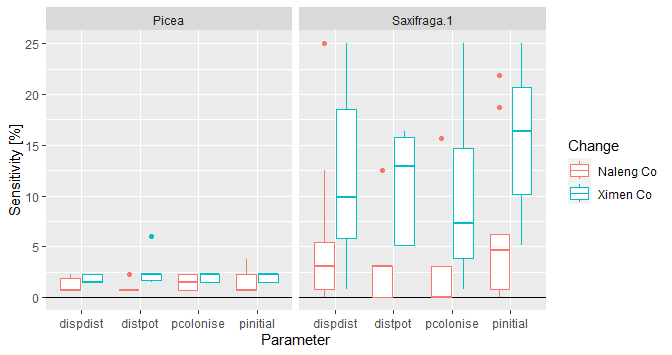
Figure S3.3. Absolute sensitivity values for two taxa (columns: *Picea* low elevation and *Saxifraga.1* high alpine) and two lakes Naleng and Ximen.

#

# **S4. Workflow of SMARC Model**


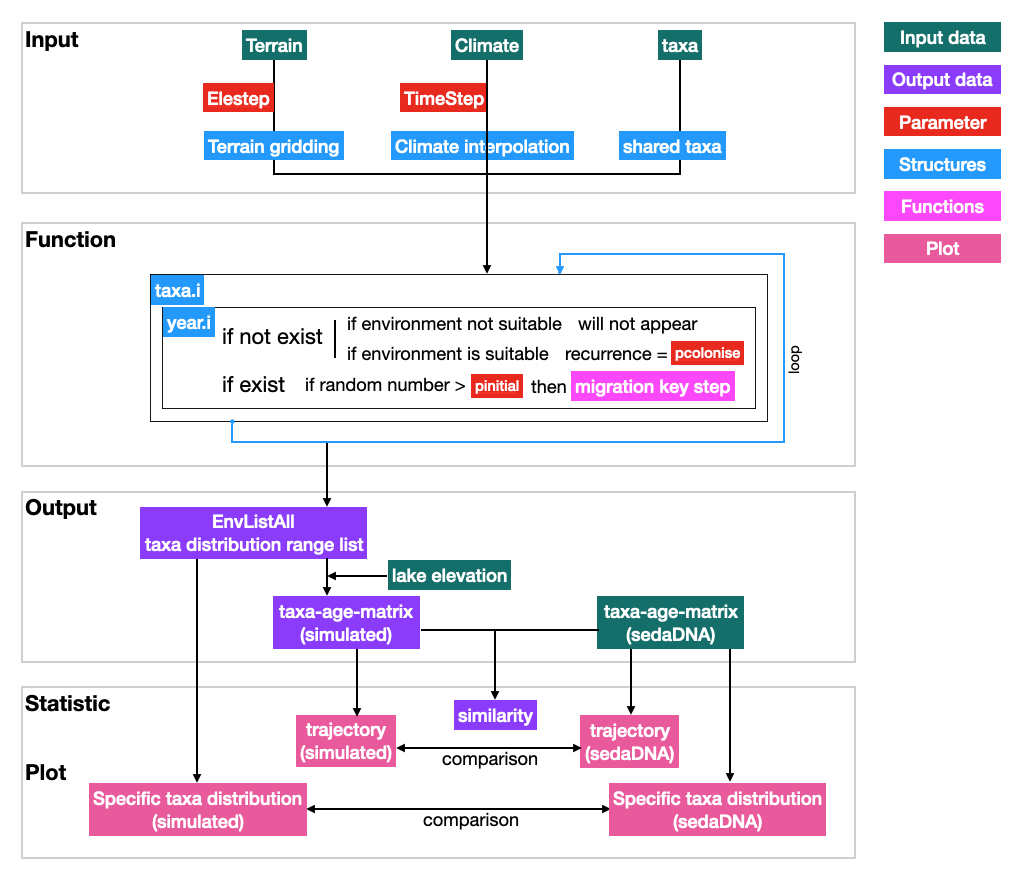


Figure S4. Workflow of SMARC model.

# **References**

Dykoski, C. et al. 2005. A high-resolution, absolute-dated Holocene and deglacial Asian monsoon record from Dongge Cave, China. – *Earth Planet. Sci. Lett*. **233**: 71–86.

Grimm, V. and Railsback, S. F. 2005. Individual-based Modeling and Ecology: – *Princeton University Press*.

Grimm, V. et al. 2006. A standard protocol for describing individual-based and agent-based models. – *Ecol. Model*. **198**: 115–126.

Grimm, V. et al. 2010. The ODD protocol: A review and first update. – *Ecol. Model*. **221**: 2760–2768.

Jarvis, A. et al. 2008. Hole-filled SRTM for the globe : version 4 : data grid.

Kruse S. et al. 2021. R_PastElevationChange: The first release of the R code R_PastElevationChange. – *Zenodo*. DOI: 10.5281/zenodo.4562675​.

Li, X. et al. 2013. Near‐surface air temperature lapse rates in the mainland China during 1962–2011. – *J. Geophys. Res. Atmospheres* **118**: 7505–7515.

Mukul, M. et al. 2017. Uncertainties in the Shuttle Radar Topography Mission (SRTM) Heights: Insights from the Indian Himalaya and Peninsula. – *Sci. Rep*. **7**: 41672.

R Core Team 2021. R: A Language and Environment for Statistical Computing. – *R Foundation for Statistical Computing*

Schoch, C. L. et al. 2020. NCBI Taxonomy: a comprehensive update on curation, resources and tools. – *Database* **2020**: baaa062.

Shakun, J. D. et al. 2012. Global warming preceded by increasing carbon dioxide concentrations during the last deglaciation. – *Nature* **484**: 49–54.

Snell, R. S. and Cowling, S. A. 2015. Consideration of dispersal processes and northern refugia can improve our understanding of past plant migration rates in North America. – *J. Biogeogr.* **42**: 1677–1688.

Svenning, J. et al. 2008. Postglacial dispersal limitation of widespread forest plant species in nemoral Europe. – *Ecography* **31**: 316–326.

Svenning, J. and Sandel, B. 2013. Disequilibrium vegetation dynamics under future climate change. – *Am. J. Bot*. **100**: 1266–1286.

Tiebel, K. et al. 2020. Restrictions on natural regeneration of storm-felled spruce sites by silver birch (*Betula pendula* Roth) through limitations in fructification and seed dispersal. – *Eur. J. For. Res*. **139**: 731–745.

Went, F. W. 1953. The effect of temperature on plant growth. – *Annu. Rev. Plant Physiol*. **4**: 347–362.
